# Extracellular volume fraction using contrast-enhanced CT is useful in differentiating intrahepatic cholangiocellular carcinoma from hepatocellular carcinoma

**DOI:** 10.3389/fonc.2023.1214977

**Published:** 2023-07-06

**Authors:** T. Honda, H. Onishi, H. Fukui, K. Yano, K. Kiso, A. Nakamoto, T. Tsuboyama, T. Ota, M. Tatsumi, S. Tahara, S. Kobayashi, H. Eguchi, N. Tomiyama

**Affiliations:** ^1^ Department of Radiology, Osaka University Graduate School of Medicine, Osaka, Japan; ^2^ Department of Radiology, Osaka Medical and Pharmaceutical University, Osaka, Japan; ^3^ Department of Pathology, Osaka University Graduate School of Medicine, Osaka, Japan; ^4^ Department of Gastroenterological Surgery, Osaka University Graduate School of Medicine, Osaka, Japan

**Keywords:** extracellular space, carcinoma, hepatocellular, cholangiocarcinoma, multidetector computed tomography, contrast media

## Abstract

**Objectives:**

To evaluate whether tumor extracellular volume fraction (fECV) on contrast-enhanced computed tomography (CT) aids in the differentiation between intrahepatic cholangiocarcinoma (ICC) and hepatocellular carcinoma (HCC).

**Methods:**

In this retrospective study, 113 patients with pathologically confirmed ICC (n = 39) or HCC (n = 74) who had undergone preoperative contrast-enhanced CT were enrolled. Enhancement values of the tumor (E_tumor_) and aorta (E_aorta_) were obtained in the precontrast and equilibrium phase CT images. fECV was calculated using the following equation: fECV [%] = E_tumor_/E_aorta_ × (100 – hematocrit [%]). fECV values were compared between the ICC and HCC groups using Welch’s *t*-test. The diagnostic performance of fECV for differentiating ICC and HCC was assessed using receiver-operating characteristic (ROC) analysis. fECV and the CT imaging features of tumors were evaluated by two radiologists. Multivariate logistic regression analysis was performed to identify factors predicting a diagnosis of ICC.

**Results:**

Mean fECV was significantly higher in ICCs (43.8% ± 13.2%) than that in HCCs (31.6% ± 9.0%, p < 0.001). The area under the curve for differentiating ICC from HCC was 0.763 when the cutoff value of fECV was 41.5%. The multivariate analysis identified fECV (unit OR: 1.10; 95% CI: 1.01–1.21; p < 0.05), peripheral rim enhancement during the arterial phase (OR: 17.0; 95% CI: 1.29–225; p < 0.05), and absence of washout pattern (OR: 235; 95% CI: 14.03–3933; p < 0.001) as independent CT features for differentiating between the two tumor types.

**Conclusions:**

A high value of fECV, peripheral rim enhancement during the arterial phase, and absence of washout pattern were independent factors in the differentiation of ICC from HCC.

## Highlights

- Extracellular volume fraction of ICC is significantly higher than that of HCC.
- Diagnosis by extracellular volume fraction has a sensitivity and specificity of 59.0% and 90.5%, respectively.
- Extracellular volume fraction is an independent factor in differentiating ICC from HCC.

## Introduction

Hepatocellular carcinoma (HCC) and intrahepatic cholangiocarcinoma (ICC) are the first and second most common primary liver malignancies, respectively (1, 2). Accurate differentiation between ICC and HCC is essential in treatment planning and for the assessment of the prognosis. Unlike HCC, surgical resection of ICC requires lymph node sampling or dissection (3–9), and radiofrequency ablation and transcatheter arterial chemoembolization are not indicated for ICC (10–13). Considering that HCC is often diagnosed noninvasively without pathologic confirmation, based on the computed tomography (CT) and/or magnetic resonance (MR) image findings in high-risk patients (14, 15), misdiagnosis of ICC as HCC on imaging studies can lead to improper treatment. Tumor biopsy is not routinely used because of its invasiveness and concern regarding procedure-related complications (16–19). There is also the possibility of misdiagnosis due to sampling errors in percutaneous liver biopsy (20–22).

According to the Liver Imaging Reporting and Data System (LI-RADS), typical ICCs are classified as LR-M (i.e., non-HCC malignancy), but atypical cases are sometimes classified as LR-4 (i.e., probably HCC) or LR-5 (i.e., definitely HCC), which decrease the diagnostic specificity for HCC (specificity of 0.84 for LR-5 as positive; 0.74 for LR-4 or LR-5 as positive) (23–26). Meanwhile, Wengert et al. (27) have reported a diagnostic algorithm using MR imaging findings that could help reliably differentiate ICC from HCC, resulting in a sensitivity and a specificity of 68.8% and 90.6%, respectively.

The estimation of the hepatic extracellular volume fraction (fECV) on equilibrium phase CT imaging is based on the direct proportionality between the concentration of contrast material and the CT attenuation. The fECV is calculated by dividing the enhancement of the regions of interest (ROIs) by the enhancement of the blood pool and then multiplying the result by the difference of 1 minus the hematocrit value during the equilibrium phase (28).

Equilibrium imaging is a technique that uses contrast agents, commonly employed in CT and MR imaging, to assess the fECV that is increased in fibrosis and other deposition processes, including amyloidosis. This method has been successfully applied with both CT and MR imaging to measure myocardial fECV (29–33) as an indirect indicator for diffuse fibrosis or to estimate histologic pancreatic fibrosis (34–36). In addition, because fECV suggests iodine levels, a method of diagnosing lymph node metastasis of papillary thyroid cancer using fECV has also been reported (37). Moreover, recent studies have demonstrated the potential of fECV to quantitatively assess diffuse fibrosis in chronic liver diseases (38–41). The fECV value reflects the proportion of extracellular interstitial space and increases with fibrosis progression, indicating the expansion of the third space (38). Therefore, fECV can accurately evaluate the degree of fibrosis in liver parenchyma. Fibrosis is often observed pathologically in ICCs (1, 42, 43), whereas it is rare in HCCs (44). Therefore, fECV may be applied to the evaluation of fibrosis of liver tumors to help distinguish ICC from HCC and is expected to be higher in ICC than that in HCC. To the best of our knowledge, no previous study has used fECV to distinguish between ICC and HCC. The aim of this study is to evaluate whether tumor fECV on contrast-enhanced CT aids in the differentiation of ICC and HCC and to prove that fECV is a variable independent of other imaging findings.

## Materials and methods

Our institutional review board approved this retrospective study and waived the requirement for informed consent.

### Patients

**Figure 1:**
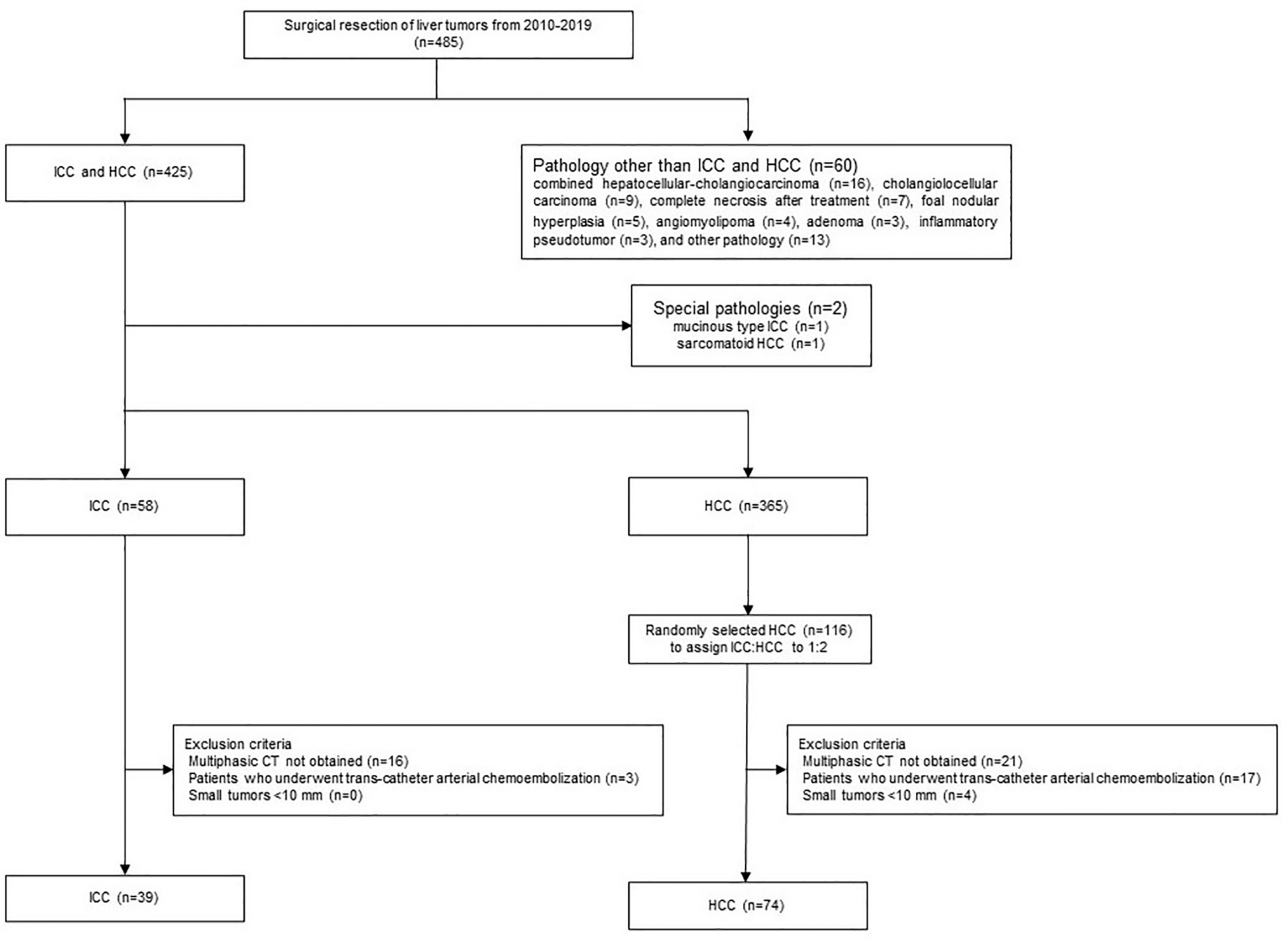
Flowchart of patient enrollment.

Candidate cases in the study were 485 patients who had undergone surgical resection of liver tumors between January 2010 and December 2019. Of these, 425 had ICCs (n = 59) or HCCs (n = 366). After excluding two cases with special pathologies such as mucinous type ICC (n = 1) or sarcomatoid HCC (n = 1), 58 had ICCs and 365 had HCCs. The final diagnoses were confirmed based on histopathological examination of the surgical specimens. As HCC cases outnumbered the ICC cases epidemiologically, 116 of the 365 HCC patients were extracted randomly to achieve an HCC/ICC ratio of 2:1. The exclusion criteria were precontrast CT and dynamic CT obtained at a different institution from our own, a tumor diameter <10 mm, and a history of preoperative transcatheter arterial chemoembolization (**Figure 1**). Finally, 39 cases in the ICC group and 74 cases in the HCC group were included. We collected and assessed the patients’ clinical data from our database and institutional medical records.

### Imaging techniques

Contrast-enhanced dynamic CT was performed using 320-channel (Aquilion ONE; Canon Medical Systems, Otawara, Japan), 256-channel (Revolution CT; GE Healthcare), or 64-channel (Discovery CT 750 HD; GE Healthcare) CT scanners. After obtaining the precontrast images, contrast agent (600 mg of iodine per kilogram of body weight) was administered intravenously with a power injector at a rate of 3–5 ml/s. Images were obtained during the arterial, portal venous, and equilibrium phases at approximately 20, 50, and 170 s, respectively, after the CT value had reached 100 HU in the aorta at the hepatic hilum level. CT images reconstructed into 5-mm slices were used in the image analyses.

### Image analysis

Quantitative analysis was performed by two radiologists (H.T. and Y.K., with 7 and 8 years’ experience in abdominal radiology, respectively) on a multimodality picture archiving and communication system (SAI workstation software, FUJIFILM, Tokyo, Japan). They were blinded to the final pathological diagnosis and all background clinical information. CT images from the ICC and HCC groups were presented randomly in a blinded manner. In patients with multiple lesions, only the largest lesion was evaluated.

CT attenuation values of the tumor and aorta were measured in images obtained before contrast agent administration and in the equilibrium phase. For each tumor, three ROIs of approximately 10 mm^2^ in area were placed on the most enhanced solid parts of the lesion, avoiding the tumor capsule, septa, and visible vessels in the lesion on the equilibrium phase. The images where each of the three ROIs were placed were not always the same slice. To enable efficient detection of fibrous characteristics, from among the three ROIs, that with the largest mean CT value in the equilibrium phase was used in the evaluation. Another ROI of the same size was placed in the same positions on the unenhanced images. For the aorta, a round ROI as large as possible was drawn within the abdominal aorta at the same level as the tumor, avoiding the aortic wall, any atheromatous plaque, and artifacts.

Tumor fECV was calculated using the following formula: fECV [%] = (100 – hematocrit [%]) × (ΔHU_tumor_/ΔHU_aorta_), where ΔHU is HU in the equilibrium phase minus HU before contrast agent administration. The hematocrit value obtained closest to the date of the CT scan was used. If no blood test was available before the CT scan, the hematocrit value of the closest day after the CT scan was used. The average fECV value of the two radiologists was used in the analysis.

### Image evaluation

Two other radiologists (K.K. and F.H., with 7 and 13 years’ experience in abdominal radiology, respectively) who were blinded to the clinicopathologic information assessed the CT image findings of the tumors independently. If there was inconsistency, consensus was achieved through discussion or referral to a third radiologist (O.H., with 25 years’ experience in abdominal radiology).

The CT images were assessed by the radiologists in accordance with important differentiating factors between ICC and HCC, which have been reported as: (1) demarcation of the tumor; (2) shape of the tumor (round, lobulated, or irregular); (3) pattern of arterial enhancement; (4) washout; (5) dilation of the bile duct; and (6) presence of tortuous tumoral vessels (24, 45). The assessment of tumor demarcation was divided into four categories: indistinct, a well-defined area of <50% of the tumor margin, a well-defined area of >50% of the tumor margin, or sharp. The arterial enhancement pattern was classified as homogeneous, heterogeneous (with mixed areas of tumor enhancement), or peripheral (with contrast enhancement at the periphery of the tumor). The washout pattern was defined as the area of the tumor that showed arterial enhancement in the arterial phase, followed by hypoattenuation compared to the surrounding hepatic parenchyma in the portal venous phase and/or equilibrium phase. Tortuous tumoral vessels were identified as enhancing vasculature within the tumor that was distinct from normal hepatic vessels and more enlarged or numerous than expected for that particular region of the liver (46).

### Statistical analysis

Statistical analysis was performed with JMP Pro version 16.2.0 (SAS Institution Japan Ltd., Tokyo, Japan). fECV values were compared between the ICC and HCC groups using Welch’s *t*-test. The tentative cutoff fECV value of the tumor that maximized the difference in the diagnosis of ICC was determined using the area under the curve (AUC), calculated by receiver operating characteristic (ROC) analysis. Subgroup analysis was also performed for patients at high risk for HCC and patients at no risk for HCC (Student’s *t*-test and Welch’s *t*-test, respectively, and ROC analysis).

Student’s *t*-test was used to compare the mean period between CT scan and the day of surgery and also the mean period between hematocrit blood test and the day of CT scan between the ICC and HCC groups. The mean areas of ROIs placed in the tumor and in the aorta were also compared by Student’s *t*-test. For both patient background and CT imaging features, differences in the numerical data of the two groups were examined by χ^2^ test or Fisher’s exact test (when n < 5). Differences in quantitative variables were evaluated by Student’s *t*-test or Welch’s *t*-test.

Univariate logistic regression analyses were performed to determine whether tumor demarcation, tumor shape, arterial enhancement pattern, washout, bile duct dilatation, and tortuous tumoral vessels were important findings in the differentiation of ICC and HCC. To identify factors predicting a diagnosis of ICC, a multivariate logistic regression analysis was performed in which four image findings considered particularly important based on the findings of previous studies (shape, arterial enhancing pattern, washout, and bile duct dilatation) as well as fECV were entered into the final model. For factors with multiple categories, the Wald test was used.

To evaluate interobserver agreement, the intraclass correlation coefficient was calculated for fECV values using the following criteria: <0.40 = poor agreement; 0.40–0.59 = fair agreement; 0.60–0.74 = good agreement; 0.75–1.00 = excellent agreement (47). Cohen’s κ coefficient was calculated for interobserver agreement in the evaluation of the CT imaging features using the following criteria: κ values of up to 0.40 were considered to indicate positive but poor agreement; 0.41–0.75, good agreement; and 0.75 or higher, excellent agreement (48).

## Results

The patient demographics and clinicopathologic characteristics in the HCC and ICC groups are listed in **Table 1**. There was no difference between the groups in terms of the mean period between the CT scan and surgery day (45.0 ± 28.7 days for ICC vs. 38.4 ± 27.8 days for HCC; *p* = 0.88) or in the mean period between the day of the hematocrit blood test and the CT scan (7.6 ± 5.8 days for ICC vs. 7.6 ± 13.3 days for HCC; *p* = 0.51).

**Table 1:** Clinicopathological characteristics in the ICC and HCC groups.

	HCC (n = 74)	ICC (n = 39)	*p* value*
**Gender, male/female**	58/16	27/12	0.360
**Age, Median (range)**	76 (58–92)	70 (44–85)	<0.0001†
**Alcohol history, Present**	32 (43.2%)	16 (41.0%)	0.844
**Diabetes, Present**	19 (25.7%)	12 (30.8%)	0.658
**HBs-Ag, Present**	32 (43.2%)	10 (25.6%)	0.101
**HCV-Ab, Present**	27 (36.5%)	4 (10.3%)	<0.01†
**AFP (ng/ml), Median (range)**	8 (0–171732)	5 (1–10413)	0.135
**PIVKA-II (mAU/ml), Median (range)**	127 (7–653168)	23 (12–66542)	0.373
**CEA (ng/ml), Median (range)**	2 (0–45)	3 (0–69)	0.0615
**CA19-9 (U/ml), Median (range)**	13.5 (0–105.9)	42 (2–41489)	0.1768
**Tumor multiplicity**			>0.99
**Single** **Multiple**	60 (81.1%) 14 (18.9%)	31 (79.5%) 8 (20.5%)	
**Primary tumor**			<0.01†
** T1** ** T2** ** T3** ** T4**	15 (20.3%) 46 (62.2%) 7 (9.5%) 6 (8.1%)	6 (15.4%) 14 (35.9%) 13 (33.3%) 6 (15.4%)	
**Lymph node metastasis, Present**	0 (0.0%)	0 (0.0%)	
**Distant metastasis, Present**	0 (0.0%)	1 (2.6%)	0.3451
**Stage**			<0.001†
** I** ** II** ** III** ** IV A** ** IV B**	15 (20.3%) 46 (62.2%) 6 (8.1%) 6 (8.1%) 1 (1.4%)	6 (15.4%) 12 (30.8%) 11 (28.2%) 3 (7.7%) 7 (18.0%)	
**Location**			0.1279
** L** ** R** ** C** ** L&R** ** L&C** ** R&C** ** L&R&C**	18 (24.3%) 48 (64.9%) 2 (2.7%) 2 (2.7%) 0 (0.0%) 1 (1.4%) 3 (4.1%)	11 (28.2%) 18 (46.2%) 2 (5.1%) 5 (12.8%) 1 (2.6%) 0 (0.0%) 2 (5.1%)	
**Pathologic differentiation**			<0.01†
** Well** ** Moderate** ** Poor** ** Unknown**	4 (5.4%) 41 (55.4%) 29 (39.2%) 0 (0.0%)	0 (0.0%) 31 (79.5%) 6 (15.4%) 2 (5.1%)	
**Stage of liver fibrosis**			<0.001†
** F0** ** F1** ** F2** ** F3** ** F4** ** Unknown**	16 (21.6%) 15 (20.3%) 17 (23.0%) 4 (5.4%) 14 (18.9%) 8 (10.8%)	20 (51.3%) 9 (23.1%) 0 (0.0%) 3 (7.7%) 5 (12.8%) 2 (5.1%)	
**Tumor size (mm), Median (range)**	29 (10–170)	37 (12–184)	0.597

*Chi-square test, Fisher’s exact test, Student’s t-test, or Welch’s t-test.

†Statistically significant.

HBs-Ag, hepatitis B surface antigen; HCV-Ab, hepatitis C virus antibody; AFP, alpha-fetoprotein; PIVKA-II, protein induced by vitamin K absence or antagonist-II; CEA, carcinoembryonic antigen; CA19-9, cancer-associated carbohydrate antigen 19-9; L, left lobe; R, right lobe; C, caudate lobe.

**Figure 2:**
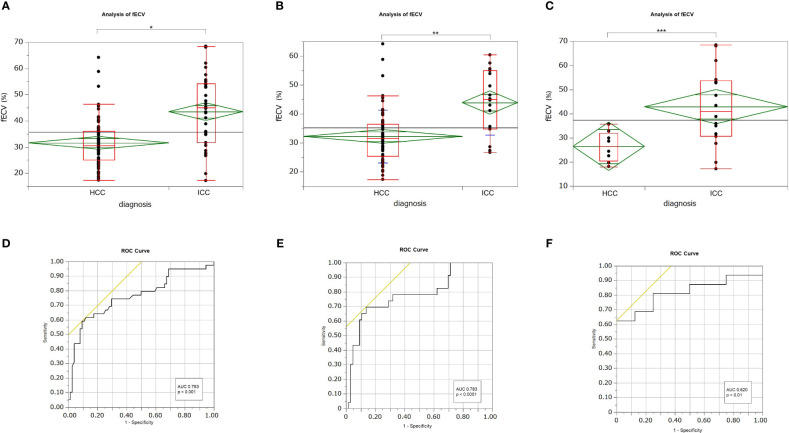
Box-and-whisker plots showing medians and ranges for extracellular volume fraction (fECV) of hepatocellular carcinoma (HCC) and intrahepatic cholangiocarcinoma (ICC) for all patients **(A)**, patients at high risk for HCC **(B)**, and patients at no risk **(C)**, respectively. The grand sample mean is represented by a horizontal black line. The boxes represent the values from the first to the third quartile. The horizontal line in each box represents the median value. The whiskers include values of 1.5 times the interquartile range. The horizontal line within each diamond is the group mean. The diamond is the confidence interval for each group. Mean fECV values were significantly higher in ICCs than those in HCCs, respectively (**A**: p < 0.001*; **B**: p < 0.001**; **C**: p < 0.01***). Receiver operating characteristic (ROC) curves showing the performance in differentiating ICCs from HCCs based on tumor fECV for all patients **(D)**, patients at high risk for HCC **(E)**, and patients at no risk **(F)**. The areas under the curves were 0.763 **(D)**, 0.783 **(E)**, and 0.820 **(F)**, respectively.

**Figure 3:**
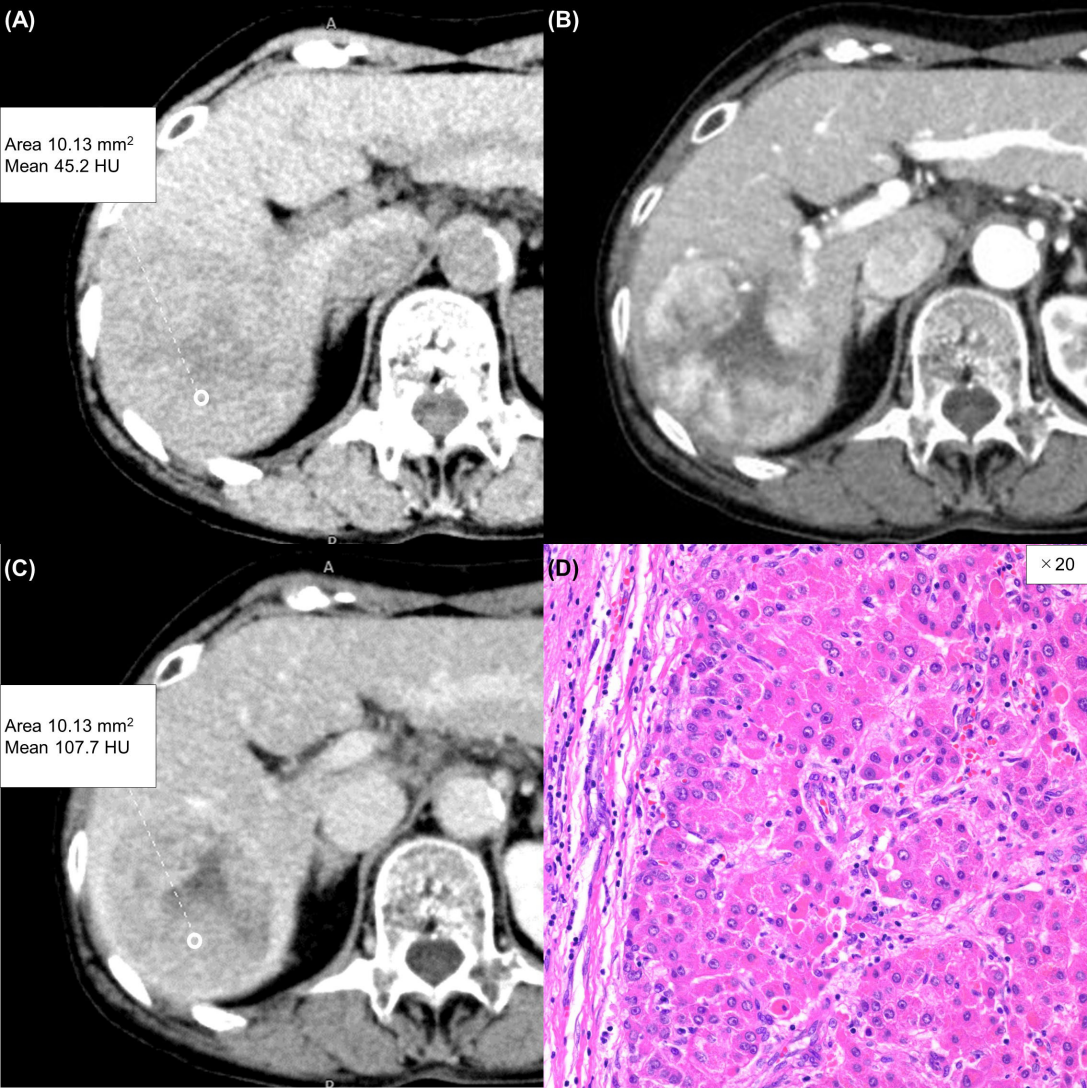
A 75-year-old woman with a typical hepatocellular carcinoma. Precontrast CT shows a round hypodense lesion in the right lobe of the liver **(A)**. The lesion shows inhomogeneous enhancement during the arterial phase **(B)** and washout during the equilibrium phase **(C)**. A round region of interest was placed in the solid part of the tumor that showed the most remarkable enhancement. Tumor extracellular volume fraction (fECV) is 33.1%, which is below the cutoff value (41.5%). No fibrosis is observed in the tumor histopathologically (**D**, ×20, hematoxylin-eosin stain).

**Figure 4:**
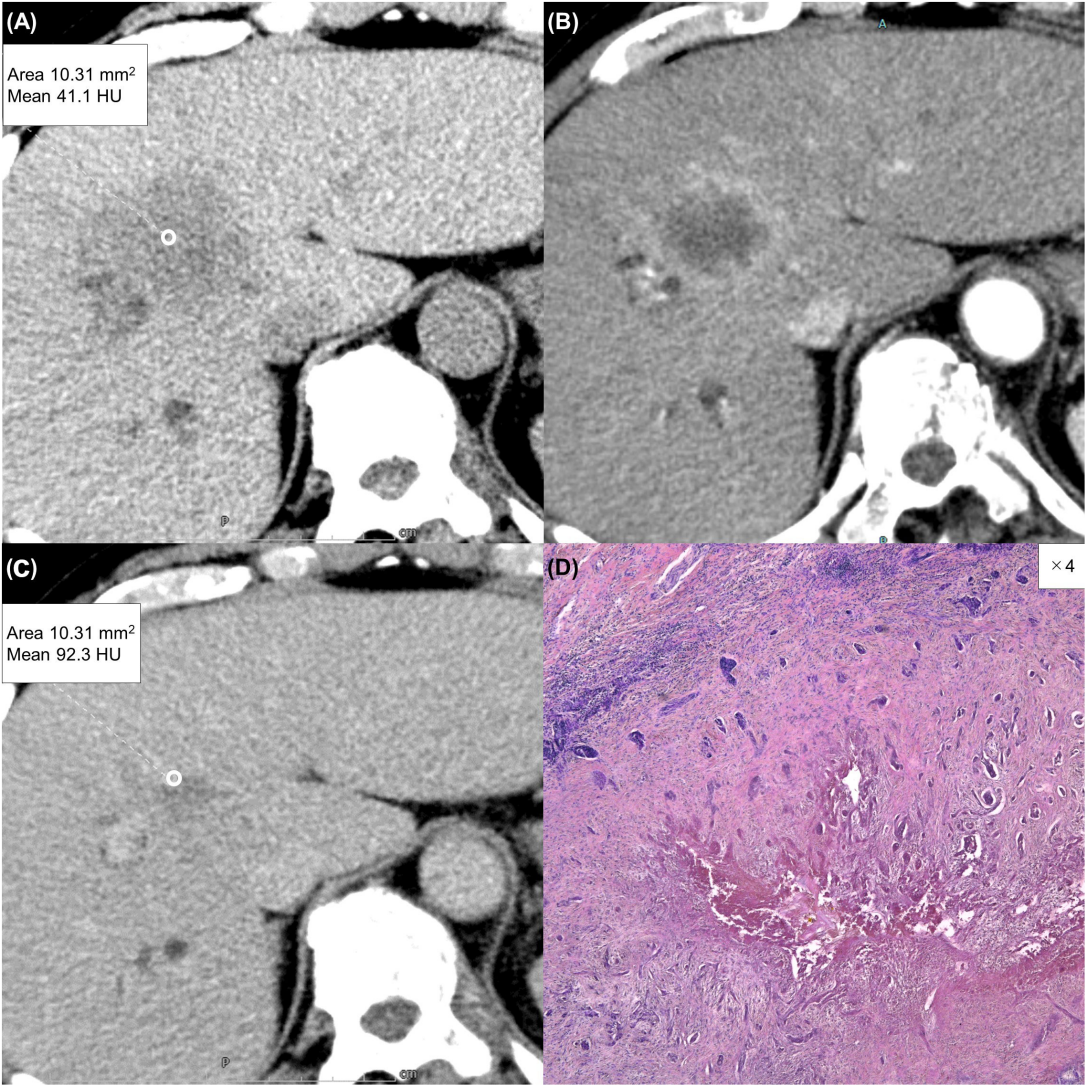
A 63-year-old man with a typical intrahepatic cholangiocarcinoma. Precontrast CT shows a hypodense lesion between the left and right lobes of the liver **(A)**. The lesion shows rim enhancement during the arterial phase **(B)** and progressive enhancement during the equilibrium phase **(C)**. Tumor extracellular volume fraction (fECV) is 52.6%, which exceeds the cutoff value (41.5%). Fibrosis is observed in the tumor histopathologically (**D**, ×4, hematoxylin-eosin stain).

Mean fECV was significantly higher in ICCs (43.8% ± 13.2%) than that in HCCs (31.6% ± 9.0%) (*p* < 0.001) (**Figures 2A**, **3**, **4**). The intraclass correlation coefficient for interobserver agreement in the fECV measurements was 0.82; 95% CI: 0.74, 0.87; well correlated. ROC analysis for differentiating ICC from HCC showed that an fECV cutoff value of 41.5% provided the maximum sum of sensitivity (59.0%) and specificity (90.5%). The AUC was 0.763 (**Figure 2D**).

Subgroup analysis revealed that in the high-risk HCC group (n = 89), the mean ECV of ICC (43.9% ± 11.1%) was significantly higher than that of HCC (32.2% ± 9.1%) (*p* < 0.0001) (**Figure 2B**). ROC analysis showed that a cutoff value of 41.1% for fECV provided the maximum sum of sensitivity (69.6%) and specificity (86.4%), and the AUC was 0.783 (**Figure 2E**). Even in the non-risk group (n = 24), the mean fECV of ICC (42.9% ± 16.0%) was significantly higher than the mean fECV of HCC (26.5% ± 6.3%) (*p* < 0.01) (**Figure 2C**). ROC analysis showed that an fECV cutoff value of 35.9% provided the maximum sum of sensitivity (62.5%) and specificity (100%), and the AUC was 0.820 (**Figure 2F**).

There was no significant difference between the two groups in terms of the mean ROI area placed in the tumor (10.2 mm^2^ in the HCC group vs. 10.2 mm^2^ in the ICC group, *p* = 0.597) or in the aorta (212 mm^2^ in the HCC group vs. 227 mm^2^ in the ICC group, *p* = 0.650).

**Table 2** lists the morphological and enhancement features in detail for each tumor type. The univariate analyses revealed fECV value, tumor shape, tumor demarcation, arterial enhancement pattern, presence of washout, and presence of bile duct dilatation as significant parameters for differentiating between the two tumors (**Table 3**). Cohen’s Kappa coefficients of interobserver agreement for each image finding are listed in **Table 4**. There was excellent agreement for all imaging findings.

**Table 2:** Morphological and enhancement features of ICC and HCC.

	HCC (n = 74)	ICC (n = 39)	*p* value*
**Shape**			<0.0001†
** Round** ** Lobulated** ** Irregular**	62 (83.8%) 7 (9.5%) 5 (6.8%)	14 (35.9%) 15 (38.5%) 10 (25.6%)	
**Demarcation**			<0.0001†
** Indistinct** ** Well-defined area <50% of tumor margin Well-defined area >50% of tumor margin Sharp**	4 (5.4%) 7 (9.5%) 5 (6.8%) 58 (78.4%)	9 (23.1%) 11 (28.2%) 8 (20.5%) 11 (28.2%)	
**Arterial enhancement pattern**			<0.0001†
** Homogeneous** ** Heterogeneous** ** Peripheral rim** ** Not enhanced**	33 (44.6%) 37 (50.0%) 4 (5.4%) 0 (0%)	7 (18.0%) 7 (18.0%) 21 (53.8%) 4 (10.3%)	
**Washout**	73 (98.6%)	8 (20.5%)	<0.0001†
**Bile duct dilatation**	5 (6.8%)	16 (41.0%)	<0.0001†
**Tortuous tumoral vessels**	14 (18.9%)	3 (7.7%)	0.1664
**fECV, mean ± SD, %**	31.6 ± 9.0	43.8 ± 13.2	<0.0001†

fECV, extracellular volume fraction.

*Chi-square test or Fisher’s exact test or Welch’s t-test.

†Statistically significant.

**Table 3:** Univariate logistic regression analysis of CT image findings.

	*p* value	Odds ratio	95% CI
Shape			
** Round**	Ref.		
** Lobulated** ** Irregular**	<0.0001† <0.001†	9.490 8.857	3.261–27.619 2.614–30.006
Demarcation			
** Indistinct** ** Well-defined area**	<0.0005† <0.0005†	11.864 8.286	3.098–45.427 2.634–26.065
<50% of tumor margin			
** Well-defined area**	<0.005†	8.436	2.322–30.645
>50% of tumor margin			
** Sharp**	Ref.		
Arterial enhancement pattern			
** Homogeneous**	0.845	1.121	0.356–3.534
** Heterogeneous**	Ref.		
** Peripheral rim**	<0.0001†	27.750	7.265–105.990
** Not enhanced**	0.991	5.753 × 10^7^	0–
**Absence of washout**	<0.0001†	282.875	33.924–2358.773
**Bile duct dilatation**	<0.0001†	9.600	3.165–29.116
**Tortuous tumoral vessels**	0.1245	0.357	0.0960–1.328
**fECV (%)**	<0.0001†	1.100 (unit odds ratio)	1.055–1.147

95% CI, 95% confidence interval; Ref., reference for categorical analysis; fECV, extracellular volume fraction.

†Statistically significant.

Unit odds ratio, Odds ratio changing for a one-unit increase.

**Table 4:** Interobserver agreement for each CT image finding.

	κ value	95% CI	Agreement
**Shape**	0.94	0.87–	excellent
**Demarcation**	0.80	0.70–0.90	excellent
**Arterial enhancement pattern**	0.92	0.86–0.98	excellent
**Washout**	0.96	0.90–	excellent
**Bile duct dilatation**	1	1	perfect
**Tortuous tumoral vessels**	0.96	0.90–1.00	excellent

κ value, Cohen’s Kappa coefficient; 95% CI, 95% confidence interval.

In the multivariate analysis, higher fECV value, peripheral rim enhancement in the arterial phase, and absence of washout pattern were independent variables predictive of ICC (*p* < 0.05, *p* < 0.05, *p* < 0.001, respectively) (**Table 5**).

**Table 5:** Multivariate analysis using multiple logistic regression model for distinguishing ICC from HCC on contrast-enhanced CT.

	*p* value*	Odds ratio	95% CI
Shape			
** Round**	Ref.	–	
** Lobulated**	0.326	4.905	0.206–117.031
** Irregular**	0.100	12.755	0.615–264.537
**Arterial enhancement pattern**	0.110		
** Homogeneous**	0.896	1.249	0.044–35.362
** Heterogeneous**	Ref.	–	
** Peripheral rim**	<0.05†	17.019	1.286–225.205
** Not enhanced**	0.997	5.002 × 10^9^	–
**Absence of washout**	0.0001†	234.908	14.032–3932.530
**Bile duct dilatation**	0.239	5.029	0.342–73.994
**fECV (%)**	<0.05†	1.097 (unit odds ratio)	1.009–1.213

*Wald test.

95% CI, 95% confidence interval; Ref., reference for categorical analysis; fECV, extracellular volume fraction.

†Statistically significant.

Unit odds ratio, Odds ratio changing for a one-unit increase.

**Figure 5:**
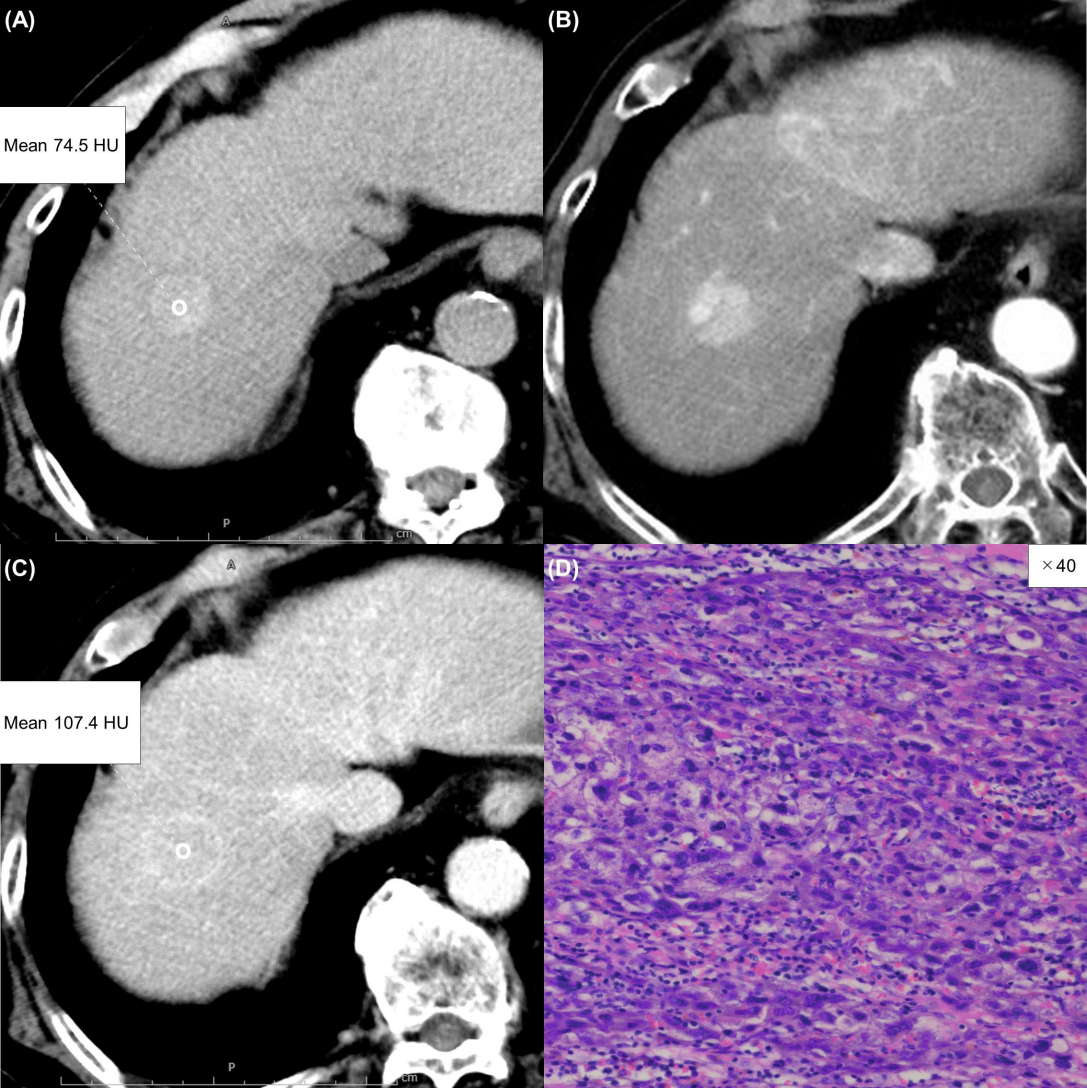
An 85-year-old man with an atypical hepatocellular carcinoma. Precontrast CT shows a round hyperdense lesion in the right lobe of the liver **(A)**. The lesion shows homogeneous enhancement during the arterial phase **(B)** and slight washout during the equilibrium phase **(C)**. Because of differences in the judgment of washout between readers 1 and 2, reader 3 made the final judgment. Tumor extracellular volume fraction (fECV) is 20.3%, which is below the cutoff value (41.5%). No fibrosis is observed in the tumor histopathologically (**D**, ×40, hematoxylin-eosin stain).

**Figure 6:**
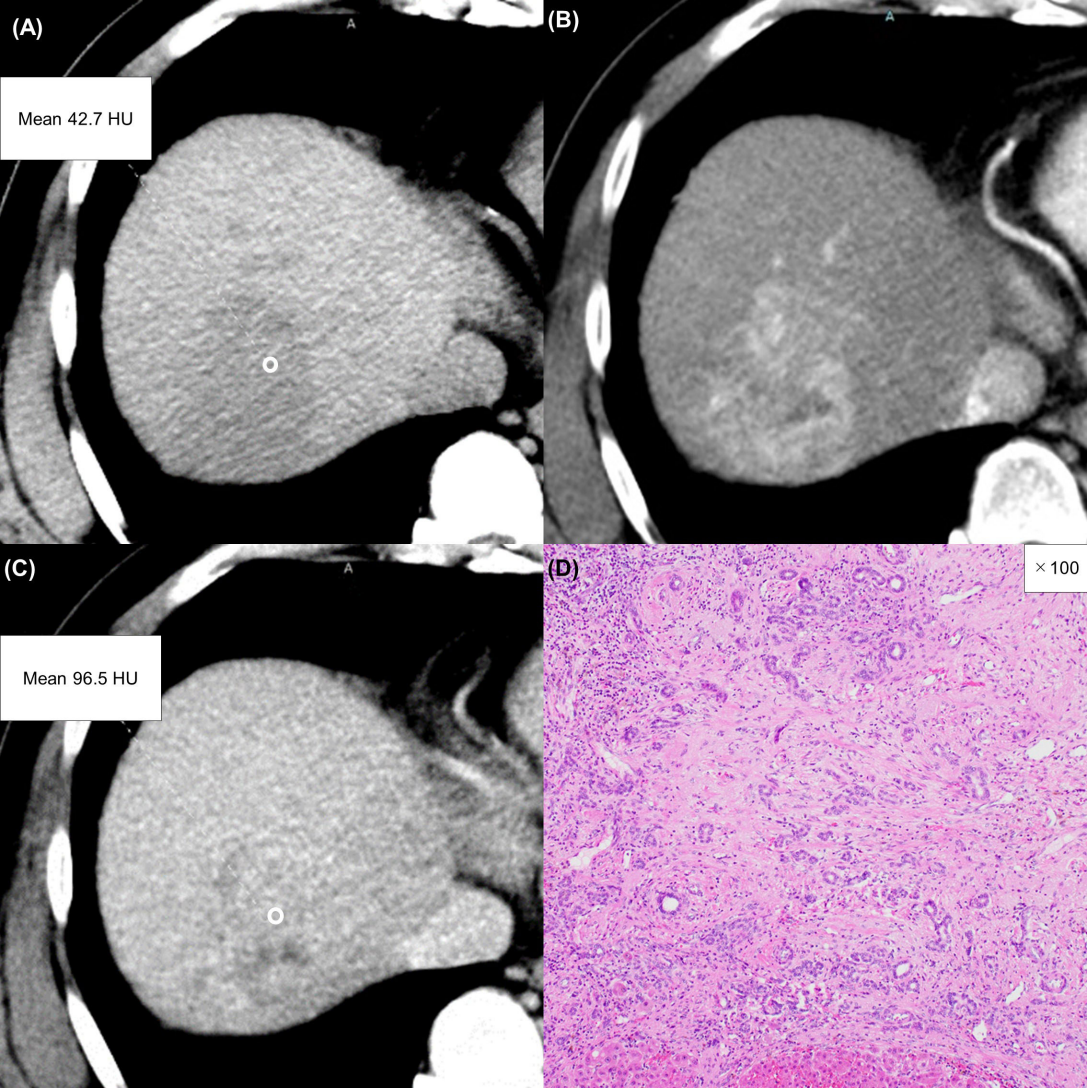
A 57-year-old man with an atypical intrahepatic cholangiocarcinoma. Precontrast CT shows a hypodense lesion in the right lobe of the liver **(A)**. The lesion shows inhomogeneous enhancement during the arterial phase **(B)** and slight washout during the equilibrium phase **(C)**. These imaging findings can lead to a misdiagnosis of hepatocellular carcinoma. A round region of interest was placed in the solid part of the tumor that showed the most remarkable enhancement. Tumor extracellular volume fraction (fECV) is 49.1%, which exceeds the cutoff value (41.5%). Fibrosis is observed in the tumor histopathologically (**D**, ×100, hematoxylin-eosin stain).

Among all HCCs, only one HCC was determined to be without washout. The fECV of this tumor was 41.7%, which is slightly higher than the cutoff value of 41.5%, indicating the possibility of ICC. There was discrepancy between the two radiologists regarding the presence or absence of washout in the two HCCs. Their fECV values (28.4% and 20.3%) were lower than the cutoff value, and both were finally determined by the third radiologist to have slight washout (**Figure 5**). Of the eight ICCs that showed washout pattern, five had fECV values (mean, 54.5%) higher than the cutoff value, indicating the possibility of ICC. In particular, one of these showed inhomogeneous arterial enhancement followed by a washout pattern characteristic of HCC and an fECV (49.6%) that exceeded the cutoff value (41.5%) (**Figure 6**). Ten ICCs that did not show washout but showed arterial enhancement characteristic of HCC had fECV values (mean, 54.2%) higher than the cutoff value.

## Discussion

ICC is well known as a representative liver tumor that is rich in fibrous tissue (1, 42, 43). In contrast, with the exception of special types, intratumoral fibrosis is rarely seen in HCC (44). In the present study, fECV values were significantly higher in ICCs than those in HCCs. This finding is thought to be caused by expansion of the third space due to proliferation of intratumoral fibrosis in ICC. Using this difference in fECV between ICC and HCC to differentiate ICC from HCC resulted in a sensitivity and a specificity of 59.0% and 90.5%, respectively. The results of the subgroup analysis suggest that fECV appears to be useful regardless of whether the patient is at risk for HCC or not. Compared to the differentiating ability using MR imaging or LI-RADS in previous articles (23, 27, 49), the use of fECV alone seems to have provided the comparable or slightly inferior differential ability. The present intraclass correlation coefficient analysis for fECV measurement by the two radiologists showed excellent agreement (0.82). Furthermore, fECV can be easily obtained without dedicated instruments or special skills.

LI-RADS is already widely used and is a method that has gained the consensus of many radiologists. Its diagnostic accuracy is also excellent, and the balance between sensitivity and specificity is well adjusted. However, LI-RADS has the disadvantage that its applicable indications are limited to patients with chronic hepatitis B and those with cirrhosis due to certain etiologies and cannot be applied to other patients. In addition, there are many imaging findings to evaluate and the algorithm is relatively complicated. Differentiation by fECV can be used regardless of the presence or absence of HCC risk, according to the results of the subgroup analysis of the current study. Furthermore, this analysis can be performed when precontrast and equilibrium phase images are available, even when arterial phase images are not available due to inappropriate timing or respiratory artifacts.

The multivariate analysis revealed enhancement pattern in the arterial phase, absence of washout finding, and high fECV as significant factors predictive of ICC. Because the fECV value is independent of enhancement pattern in the arterial phase and the washout findings, adding the fECV value to conventional image evaluation may potentially improve diagnostic accuracy for ICC and HCC. Adding fECV evaluation to current LI-RADS decisions as an ancillary feature might also improve diagnostic accuracy for HCC.

Areas of delayed or prolonged enhancement inside a liver tumor on CT are widely considered to correspond histopathologically to fibrotic stroma (42, 43). The cause of this appearance has been reported to be the slow wash-in and washout of the extravascular flux of iodinated contrast material in fibrous tissue (50). From this fact, it had been inferred that the fECV value might correlate to the washout finding and these might not be independent factors, as both demonstrate the behavior of contrast medium in the fibrous tissue of a tumor during the equilibrium phase. However, the present multivariate analysis revealed washout finding and fECV value as independent factors for the following reason. Unlike washout pattern, the fECV value is not affected by the CT value of the surrounding liver parenchyma. Also, unlike washout pattern, the fECV value is affected by the CT value on the precontrast image. We consider that these differences enable the fECV value and washout finding to be used as independent factors. There are two possible causes of washout despite high fECV, or *vice versa*. The first of these is fatty liver and tumor fatty deposits (51). The determination of washout by LI-RADS indicates that the mass has visually lower attenuation than the surrounding liver parenchyma during the portal venous and/or equilibrium phases. Therefore, visual contrast is affected when the CT value of the hepatic parenchyma is low or when the CT value of the tumor is low. Similarly, visual contrast is also affected in pathologies that cause an increase in CT values, such as metal deposits in hepatic parenchyma or tumors. Visual contrast can additionally be affected when the degree of contrast of the liver parenchyma is influenced by body size or the amount of contrast agent administered. The calculated fECV value appears to indicate the contrast effect in a tumor independently of the visual contrast between the tumor and the liver parenchyma. The second cause is that even if a tumor shows partial washout (determined to have washout), the fECV can be high in tumors that have a partial high contrast portion in the equilibrium phase.

To accurately detect the characteristics of fibrosis, the present ROIs were set and measured at portions of the tumor where fibrosis was most likely to be rich, i.e., in areas with a high contrast enhancement during the equilibrium phase. We also considered contouring the entire tumor as the ROI, but this method was not adopted due to the risk of contamination by necrotic areas adversely affecting the evaluation of fibrosis. We were concerned that the subjectivity of the measurer could affect reproducibility when the ROIs were placed in areas that appeared visually to have high contrast enhancement; however, the present interobserver agreement was excellent (intraclass correlation coefficient, 0.82). This result indicates adequate reproducibility.

This retrospective cohort study has several limitations. First, CT values during the equilibrium phases can be influenced by various factors, including the type of CT scanner, scan protocol, and contrast enhancement protocol (52, 53). As the present CT images were obtained by three different scanners, differences among the scanners may not have had a significant influence on CT values. Second, our study did not include other hepatic tumors such as combined hepatocellular cholangiocarcinoma. Further investigations are required that include other entities. Finally, in the present study, we evaluated only arterial rim enhancement, washout pattern, and peripheral bile duct dilation as representative imaging findings in the multivariate analysis due to the small number of cases. We would like to emphasize that other findings, including enhancing capsule, are also important findings in the differentiation of HCC and ICC.

In conclusion, multivariate analysis identified a higher value of fECV, peripheral rim enhancement in the arterial phase, and absence of washout pattern as independent factors for distinguishing ICC from HCC. The addition of fECV analysis to the evaluation of imaging features on contrast-enhanced CT could potentially improve the accuracy of differentiating between ICC and HCC.

## Data availability statement

The raw data supporting the conclusions of this article will be made available by the authors, without undue reservation.

## Ethics statement

The studies involving human participants were reviewed and approved by Graduate School of Medicine, Osaka University. Written informed consent for participation was not required for this study in accordance with the national legislation and the institutional requirements.

## Author contributions

TH, HO, and NT contributed to conception and design of the study. HF, KY, KK, AN, TT, TO, MT, ST, SK, and HE organized the database. TH and HO performed the statistical analysis, wrote the manuscript. All authors contributed to manuscript revision, read, and approved the submitted version.
