# Erratum: Extracellular volume fraction using contrast-enhanced CT is useful in differentiating intrahepatic cholangiocellular carcinoma from hepatocellular carcinoma

**DOI:** 10.3389/fonc.2023.1282057

**Published:** 2023-09-05

**Authors:** Frontiers Production Office

**Affiliations:** Frontiers Media SA, Lausanne, Switzerland

**Keywords:** extracellular space, carcinoma, hepatocellular, cholangiocarcinoma, multidetector computed tomography, contrast media

Due to a production error, there was a mistake in **Figure 4** as published. In the figure, subfigure (C) is incorrectly labeled as (D). The corrected **Figure 4** appears below. The publisher apologizes for this mistake.

**Figure 4:**
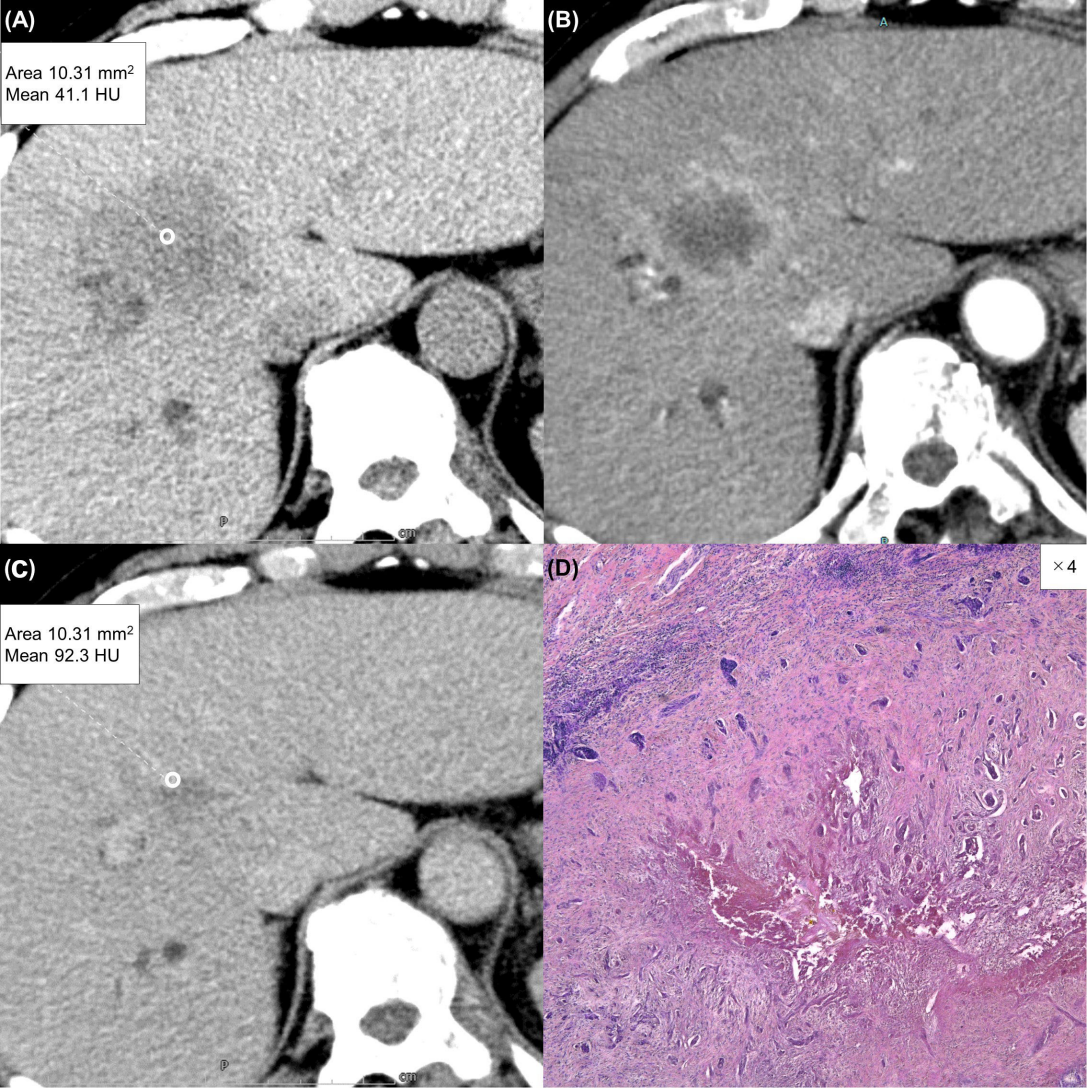
A 63-year-old man with a typical intrahepatic cholangiocarcinoma. Precontrast CT shows a hypodense lesion between the left and right lobes of the liver **(A)**. The lesion shows rim enhancement during the arterial phase **(B)** and progressive enhancement during the equilibrium phase **(C)**. Tumor extracellular volume fraction (fECV) is 52.6%, which exceeds the cutoff value (41.5%). Fibrosis is observed in the tumor histopathologically (**D**, ×4, hematoxylin-eosin stain).

The original version of this article has been updated.

